# Activin and BMP Signaling Activity Affects Different Aspects of Host Anti-Nematode Immunity in *Drosophila melanogaster*

**DOI:** 10.3389/fimmu.2021.795331

**Published:** 2021-12-22

**Authors:** Yaprak Ozakman, Dhaivat Raval, Ioannis Eleftherianos

**Affiliations:** Infection and Innate Immunity Laboratory, Department of Biological Sciences, The George Washington University, Washington, DC, United States

**Keywords:** *Drosophila*, innate immunity, *Heterorhabditis*, *Photorhabdus*, TGF-ß

## Abstract

The multifaceted functions ranging from cellular and developmental mechanisms to inflammation and immunity have rendered TGF-ß signaling pathways as critical regulators of conserved biological processes. Recent studies have indicated that this evolutionary conserved signaling pathway among metazoans contributes to the *Drosophila melanogaster* anti-nematode immune response. However, functional characterization of the interaction between TGF-ß signaling activity and the mechanisms activated by the *D. melanogaster* immune response against parasitic nematode infection remains unexplored. Also, it is essential to evaluate the precise effect of entomopathogenic nematode parasites on the host immune system by separating them from their mutualistic bacteria. Here, we investigated the participation of the TGF-ß signaling branches, activin and bone morphogenetic protein (BMP), to host immune function against axenic or symbiotic *Heterorhabditis bacteriophora* nematodes (parasites lacking or containing their mutualistic bacteria, respectively). Using *D. melanogaster* larvae carrying mutations in the genes coding for the TGF-ß extracellular ligands Daw and Dpp, we analyzed the changes in survival ability, cellular immune response, and phenoloxidase (PO) activity during nematode infection. We show that infection with axenic *H. bacteriophora* decreases the mortality rate of *dpp* mutants, but not *daw* mutants. Following axenic or symbiotic *H*. *bacteriophora* infection, both *daw* and *dpp* mutants contain only plasmatocytes. We further detect higher levels of *Dual oxidase* gene expression in *dpp* mutants upon infection with axenic nematodes and *Diptericin* and *Cecropin* gene expression in *daw* mutants upon infection with symbiotic nematodes compared to controls. Finally, following symbiotic *H. bacteriophora* infection, *daw* mutants have higher PO activity relative to controls. Together, our findings reveal that while *D. melanogaster* Dpp/BMP signaling activity modulates the DUOX/ROS response to axenic *H. bacteriophora* infection, Daw/activin signaling activity modulates the antimicrobial peptide and melanization responses to axenic *H. bacteriophora* infection. Results from this study expand our current understanding of the molecular and mechanistic interplay between nematode parasites and the host immune system, and the involvement of TGF-ß signaling branches in this process. Such findings will provide valuable insight on the evolution of the immune role of TGF-ß signaling, which could lead to the development of novel strategies for the effective management of human parasitic nematodes.

## Introduction

Transforming growth factor (TGF-ß) signaling is an evolutionary conserved signaling pathway among metazoans (1). Having components present in all animals studied to date, TGF-ß signaling has been regarded as a key pathway in metazoan evolution and in transition to multicellularity (1, 2). In addition, its role has been implicated in various biological processes ranging from development to metabolism (3). Compared to vertebrates, TGF-ß signaling in *Drosophila melanogaster* comprises fewer representatives of each signaling component, yet still regulates diverse functions including axis formation, body patterning, and morphogenesis (4–7). Activin and bone morphogenetic protein (BMP) are the two signaling branches of the *D. melanogaster* TGF-ß pathway, which are characterized by extracellular ligands, type I and type II receptors, and intracellular transducers (8). Signaling is initiated by the binding of ligands to transmembrane receptor complex of serine/threonine kinases, which in turn phosphorylates transcription factors that regulate the activation of downstream genes (9).

In addition to its contribution to wounding response and anti-bacterial immunity, the role of TGF-ß signaling has been implicated in anti-nematode immunity in *D. melanogaster* (10–14). In particular, gene transcript levels of the ligands Daw (activin branch) and Dpp (BMP branch) are upregulated following infection with the parasitic nematodes, *Heterorhabditis gerrardi* and *Heterorhabditis bacteriophora* in adult flies (12). Inactivation of *dpp* leads to increased survival ability and induction of humoral immunity against *H. bacteriophora* infection (11). Also, in response to *H. gerrardi* infection, *daw* mutants have decreased expression of *Dual oxidase* (*Duox*) compared to their background controls (14). However, detailed functional characterization of the interaction between TGF-ß signaling activity and cellular, humoral, and melanization responses in *D. melanogaster* against parasitic nematode infection remains incomplete.

The entomopathogenic nematode (EPN) *H. bacteriophora* and its mutualistic bacterium *P. luminescens* constitute an excellent model to elucidate the interaction between TGF-ß signaling and host anti-nematode immunity. The nematodes and their bacteria, together or separately, can infect and kill a variety of insect species (15). Infective juveniles (IJs), the only free-living stage of the nematodes, enter the insect hemocoel either through natural openings or by using their special appendage to penetrate through the cuticle (16). Upon entry, IJs regurgitate their *Photorhabdus* bacteria into the hemolymph to spread into the host and overcome the insect immune response (17, 18). Once the insect dies and resources in the cadaver are depleted, IJs emerge from the carcass to search for new prey (15) *D. melanogaster* is not a natural host of *H. bacteriophora* (19). However, previous studies have established methods for infecting *D. melanogaster* larvae and adult flies with *Heterorhabditis* nematodes, which can develop efficiently in both stages of the host (19, 20).

Although previous studies have shown that TGF-ß signaling interacts with the *D. melanogaster* larval and adult immune response during infection with the *Heterorhabditis*-*Photorhabdus* complex, it is crucial to elucidate the precise effect of the parasites on the host immune system by separating them from their mutualistic bacteria (11–14). To this end, we investigated the participation of the two TGF-ß signaling branches, activin and BMP, in host immune function against axenic (lacking *P. luminescens*) or symbiotic (containing *P. luminescens*) *H. bacteriophora* nematodes. Using *D. melanogaster* larvae carrying mutations in the genes coding for the TGF-ß extracellular ligands Daw and Dpp, we explored modifications in cellular, humoral, and melanization responses during infection with these pathogens.

In this study, we show that BMP signaling activity promotes survival to *D. melanogaster* larvae when infected by symbiotic *H. bacteriophora* but increases mortality to larvae infected by axenic nematodes. Also, following axenic *H. bacteriophora* infection, BMP signaling activity leads to reduced *DUOX* expression and increased *Diptericin* and *Drosomycin* antimicrobial gene expression. Similarly, activin signaling activity upregulates *Drosomycin* expression against axenic nematode infection but downregulates *Diptericin* and *Cecropin* expression against symbiotic nematode infection. Finally, we demonstrate that in response to symbiotic *H. bacteriophora* infection, activin signaling activity lowers PO activity in *D. melanogaster* larvae. Taken together our findings provide a detailed characterization of the *D. melanogaster* activin and BMP signaling activity regulation in relation to host immune function against parasitic nematodes deficient of their mutualistic bacteria. The information garnered from these results set the stage for elucidating the evolution of the immune role of TGF-ß signaling against parasitic nematode infection and contribute to the effective use of *Heterorhabditis* nematodes as models for human parasitic diseases.

## Materials and Methods

### Fly and Nematode Stocks

All flies were reared on Bloomington Drosophila Stock Center cornmeal food (Labexpress) supplemented with yeast (Carolina Biological Supply), maintained at 25°C, and a 12:12-h light:dark photoperiodic cycle. Larvae carrying a spontaneous dpp^s1^ mutation (strain 397, Bloomington, IL, USA) and carrying P-bac insertion Pbac{XP}daw05680 (strain d05680, Exelixis, Boston, MA, USA) were used, as previously described (14). Line w*^1118^* (strain 3605, Bloomington, IL, USA) was used as the background control. Infective juveniles (IJs) of *H. bacteriophora* strain TT01 were amplified in larvae of the wax moth *Galleria mellonella* using the water trap technique (21). To generate axenic *H. bacteriophora* nematodes lacking their *P. luminescens* bacteria, a previously established protocol was followed (22). Prior to experiments, axenic nematodes were surface sterilized in 5% bleach solution and rinsed five times with water to remove the bleach residue. Nematodes were used 1-4 weeks after collection.

### Larval Infection and Survival Assay

Infections were carried out using 96-well plates containing 100 μL of 1.25% agarose in each well. The suspension of axenic or symbiotic *H. bacteriophora* IJs (100 nematodes in 10 μL of sterile distilled water) was added to a single larva (late second to third instar) in each well. Sterile distilled water (10 μL) served as uninfected control. The plate was covered with a sealing film (USA Scientific, Ocala, FL, USA) and air holes were poked for ventilation. Plates were kept in the dark at room temperature for 24 h. At the 24-h time point, larvae were collected and frozen at −80°C, or immediately used in experiments. The survival of larvae kept in nematode solution or in sterile distilled water was estimated at 12-h intervals and up to 60 h. The survival experiments were replicated three times.

### Gene Expression Analysis

RNA was extracted from 4-5 *D. melanogaster* larvae (late second to third instar) using Trizol reagent (Ambion, Life Technologies) and reverse transcription was performed using High-Capacity cDNA Reverse Transcription Kit (Applied Biosystems) according to the manufacturers’ instructions. Real time PCR experiments were conducted in a CFX96 Real-Time System, C1000 Thermal Cycler (Bio-Rad) with gene-specific primers (**Table 1**) using GreenLink qPCR Mix (BioLink). Each experiment was run in technical triplicates and repeated three times.

**Table 1:** Primers and their sequences used in quantitative qPCR experiments.

Gene	Accession Number	Primer	Sequence (5’ - 3’)	Tm (°C)
***NOS***	CG6713	Forward	AACGTTCGACAAATGCGCAA	60
		Reverse	GTTGCTGTGTCTGTGCCTTC	
***DUOX***	CG3131	Forward	ACGTGTCCACCCAATCGCACGAG	60
		Reverse	AAGCGTGGTGGTCCAGTCAGTCG	
***PPO1***	CG5779	Forward	CAACTGGCTTCGTTGAGTGA	60
		Reverse	CGGGCAGTTCCAATACAGTT	
***PPO2***	CG8193	Forward	CCCGCCTATACCGAGA	59
		Reverse	CGCACGTAGCCGAAAC	
***PPO3***	CG42640	Forward	GGCGAGCTGTTCTACT	58
		Reverse	GAGGATACGCCCTACTG	
***Diptericin***	CG12763	Forward	GCTGCGCAATCGCTTCTACT	60
		Reverse	TGGTGGAGTTGGGCTTCATG	
***Cecropin***	CG1365	Forward	TCTTCGTTTTCGTCGCTCTC	57
		Reverse	CTTGTTGAGCGATTCCCAGT	
***Drosomycin***	CG10810	Forward	GACTTGTTCGCCCTCTTCG	60
		Reverse	CTTGCACACACGACGACAG	
***Metchnikowin***	CG8175	Forward	TCTTGGAGCGATTTTTCTGG	56
		Reverse	AATAAATTGGACCCGGTCTTG	
***RpL32***	CG7939	Forward	GATGACCATCCGCCCAGCA	60
		Reverse	CGGACCGACAGCTGCTTGGC	

### Hemocyte Assays

Following axenic or symbiotic *H. bacteriophora* infection, 10 *D. melanogaster* larvae (late second to third instar) were bled into 30 μl of 2.5× protease inhibitor cocktail (Sigma). Hemolymph samples were loaded on a hemocytometer and total numbers and aggregates of cells were counted using 40× magnification on a compound microscope (Olympus CX21). Clumps of two or more hemocytes were considered as aggregates and each clump was counted as one aggregate. In each experiment five technical replicates were used, and each experiment was repeated three times.

### Hemocyte Staining and Microscopy

To distinguish the different types of hemocytes following infection with axenic or symbiotic *H. bacteriophora*, six *D. melanogaster* larvae (late second to third instar) were washed in 1xPBS and bled into 30 μl of 1x PBS on a poly-lysine coated microscope slide which was air dried for 20 min. Hemocytes were fixed in 4% paraformaldehyde for 15 min, washed with 1xPBS, permeabilized in PBST (0.3% Triton in 1x PBS) for 20 min, and washed again in 1x PBS. Hemocytes were blocked in PBSTB (8% BSA in PBST) for 15 min, washed in 1x PBS, and then incubated with diluted phalloidin TRITC (1:100) for 20 min before a final wash in 1x PBS. Using ProLong^®^ Gold Antifade reagent with DAPI, hemocytes were mounted and stored at 4°C until imaging. Hemocytes were visualized using Zeiss LSM 510 confocal microscope and their types were distinguished based on morphology, as previously described (23). The experiment was repeated three times.

### Nitric Oxide and Aconitase Assays

To assess levels of Nitric Oxide (NO) and aconitase activity, six late second to third instar *D. melanogaster* larvae were collected 24-h post-infection with axenic or symbiotic *H. bacteriophora*. For NO estimation, larvae were homogenized in PBS and centrifuged at 10,000 x g for 10 min at 4°C. The resultant supernatant was used to quantify proteins as previously described and then mixed 1:1 with Griess reagent (Sigma) (1). Absorbance was measured at 595 nm using a plate reader (BioTek). To calculate NO levels, a silver nitrite standard curve was used. For the estimation of aconitase activity, larvae were homogenized in aconitase assay buffer and processed following the manufacturer’s instructions (MAK051-1KT; Sigma). Absorbance was measured at 450 nm and levels of aconitase activity were calculated from an isocitrate standard curve. Experiments were conducted in technical duplicates and repeated three times.

### Phenoloxidase Assay

To collect hemolymph following infection with axenic or symbiotic *H. bacteriophora*, 10 larvae from each *D. melanogaster* line were bled into 30 μl of 2.5x protease inhibitor. Hemolymph samples were added to Pierce^®^ Spin Columns and spun at 4°C and 13,000 rpm for 10 min. Protein concentrations were measured using the Pierce™ BCA Protein Assay Kit (Thermo Fisher Scientific). A mixture containing 15 μg of protein, 5 mM CaCl_2_, and 2.5× protease inhibitor was added to 160 μl of fresh L-DOPA solution (in phosphate buffer, pH 6.6) in a clear microplate well. Absorbance values were measured at 29°C and 492 nm for 60 min. Absorbance of the blank was subtracted from the absorbance of each sample. Each experiment was repeated three times.

### Assessment of Melanization

To observe the ability of hemolymph to melanize following infection with axenic or symbiotic *H. bacteriophora*, hemolymph samples were collected from 10 larvae of each *D. melanogaster* line in 1xPBS, added to a Pierce^®^ Spin Column and spun at 4°C and 13,000 rpm for 10 min. Hemolymph plasma samples were transferred to a 96 well microtiter plate. After 3 h incubation at room temperature, the presence of melanization in the wells was noted.

### Statistical Analysis

GraphPad Prism 8 was used for data plotting and statistical analyses. Statistics for the results from the survival experiments were carried out using log-rank (Mantel-Cox) test. One-way analysis of variance (ANOVA) and Tukey *post-hoc* tests were used for analyzing the results from the rest of the experiments.

## Results

### BMP Branch Activity Promotes Survival of *D. melanogaster* Larvae Upon Symbiotic *H. bacteriophora* Infection

**Figure 1:**
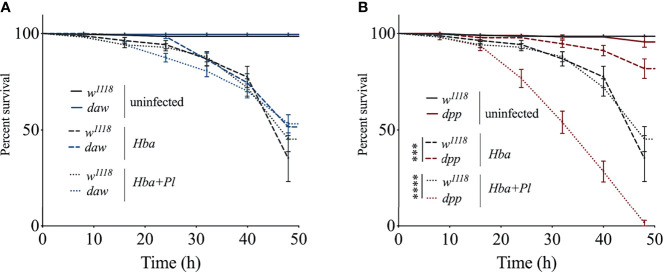
Survival analysis of *Drosophila melanogaster* TGF-ß mutant larvae upon infection with axenic (*Hba*) or symbiotic (*Hba*+*Pl*) *Heterorhabditis bacteriophora*. *Daw* and *dpp* mutant larvae and their background controls (*w^1118^*) were infected with either *H. bacteriophora* axenic or symbiotic nematodes. Treatment with water served as negative control (uninfected). Larval survival was estimated every 12 h and up to 48 h after infection. Significance levels were assessed using log-rank (Mantel-Cox) test. Survival ability of *daw* **(A)** and *dpp* **(B)** mutants following infection with axenic or symbiotic *H. bacteriophora* (***p = 0.0002, ****p < 0.0001).

To understand whether activin and BMP TGF-ß signaling branches contribute to the survival ability of *D. melanogaster* larvae against infection with *H. bacteriophora* containing or lacking their mutualistic bacteria, we challenged *daw* and *dpp* mutants with either type of nematode to assess their time-course survival rate following infection. For this, we monitored larval survival every 12 h and up to 48 h post nematode infection. We did not observe any significant differences between *daw* mutants and their background controls (*w^1118^*) following axenic or symbiotic *H. bacteriophora* infection (**Figure 1A**). However, we found that while *dpp* mutants showed higher survival rates upon axenic *H. bacteriophora* infection, their survival ability to symbiotic nematode infection decreased compared to *w^1118^* controls (**Figure 1B**). These results indicate that the BMP signaling activity in *D. melanogaster* larvae promotes survival against symbiotic *H. bacteriophora* infection but limits survival against axenic *H. bacteriophora* infection.

### Activin and BMP Signaling Activity Do Not Participate in the *D. melanogaster* Larval Cellular Immune Response to *H. bacteriophora* Infection

**Figure 2:**
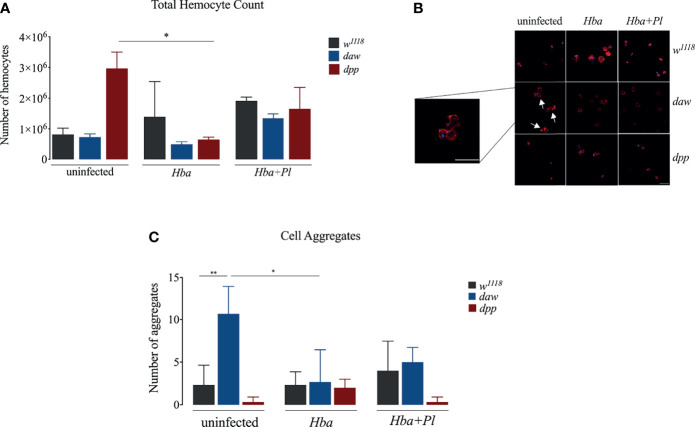
Cellular immune response of *Drosophila melanogaster* TGF-ß mutant larvae against axenic (*Hba*) or symbiotic (*Hba*+*Pl*) *Heterorhabditis bacteriophora* nematode infection. **(A)** Total number of circulating hemocytes in *D. melanogaster daw* and *dpp* mutant larvae and their background controls (*w^1118^*) upon infection with either axenic or symbiotic nematodes. Hemolymph samples were collected from 10 larvae at 24 h following infection and the number of circulating hemocytes was assessed using a hemocytometer under a light microscope (*p = 0.0343). **(B)** Confocal microscopy images of hemocytes stained with Phalloidin-TRITC (red) and DAPI (blue) in *D. melanogaster daw* and *dpp* mutants and their background controls (*w^1118^*) following axenic or symbiotic *H. bacteriophora* nematode infection. Scale bar is 20 μm. White arrows indicate hemocyte aggregates. **(C)** Assessment of the number of hemocyte aggregates in *D. melanogaster daw* and *dpp* mutant larvae and their background controls (*w^1118^*) following *H. bacteriophora* axenic or symbiotic nematode infection (*p = 0.0123, **p = 0.0086). Significance levels were assessed using one-way analysis of variance (ANOVA).

The cellular immune response is mediated by the hemocytes and forms an integral part of the *D. melanogaster* host defense because it acts in conjunction with humoral immune mechanisms to oppose infection (24, 25). Previous studies have linked the number of hemocytes to immune system capacity in *D. melanogaster* (26–29). To determine whether activin and BMP signaling activity modulate the number of hemocytes in *D. melanogaster* larvae responding to parasitic nematode infection, we counted the number of circulating hemocytes in *daw* and *dpp* mutants following infection with axenic or symbiotic *H. bacteriophora*. Of note, here we focused only on circulating hemocytes, but there is a possibility that some of the sessile hemocytes may have become circulating to act on the anti-nematode response. We found that the number of hemocytes was not significantly affected in TGF-ß mutants when infected with either type of nematode compared to their background controls. However, hemocyte numbers decreased in *dpp* mutants following infection with axenic *H. bacteriophora* relative to uninfected *dpp* individuals suggesting a link between hemocyte proliferation and BMP signaling activity (**Figure 2A**). Wasp parasitization in *D. melanogaster* larvae induces the differentiation of lamellocytes, which are specialized type of hemocytes (29–32). Previous evidence suggests that infection with the EPN *Steinernema carpocapsae* also leads to lamellocyte differentiation in *D. melanogaster* wild-type larvae (23). To elucidate whether activin and BMP signaling branches in *D. melanogaster* larvae are involved in the differentiation of lamellocytes during *H. bacteriophora* infection, we stained hemocytes in *daw* and *dpp* mutants and their *w^1118^* background controls with phalloidin-TRITC and DAPI and visualized them using confocal microscopy (**Figure 2B**). We only detected plasmatocytes in either TGF-ß mutant following infection with axenic or symbiotic *H. bacteriophora* nematodes. Interestingly, we noticed the formation of a higher number of hemocyte aggregates in uninfected *daw* mutants compared to their background controls. To quantitively assess whether inactivating TGF-ß signaling branches leads to changes in hemocyte aggregation in the context of nematode infection, we counted the number of hemocyte aggregates in *daw* and *dpp* mutants following infection with axenic or symbiotic *H. bacteriophora*. Consistent with the initial observation, we found that in the absence of infection, there was a higher number of hemocyte aggregates in *daw* mutants compared to those detected in *w^1118^* individuals. In addition, following axenic *H. bacteriophora* infection, the number of cell aggregates decreased in *daw* mutant larvae compared to their uninfected counterparts (**Figure 2C**). These findings suggest that activin and BMP signaling activity do not affect cellular immune processes in *D. melanogaster* larvae, such as total hemocyte numbers, differentiation of lamellocytes, and formation of hemocyte aggregates upon *H. bacteriophora* infection.

### BMP Signaling Activity in *D. melanogaster* Larvae Lowers Expression of *DUOX* Upon Axenic *H. bacteriophora* Infection

**Figure 3:**
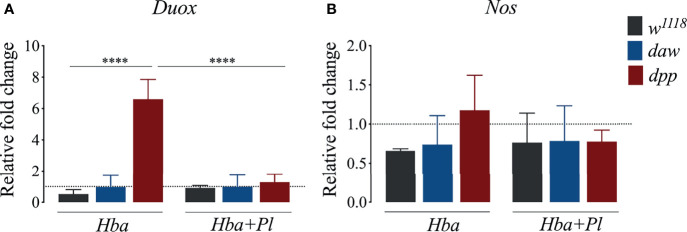
Quantitative PCR analysis of *dual oxidase* (*DUOX*) **(A)** and *nitric oxide synthase* (*NOS*) **(B)** expression in *Drosophila melanogaster daw* and *dpp* mutant larvae and their background controls (*w^1118^*) responding to infection by either axenic (*Hba*) or symbiotic (*Hba* + *Pl*) *Heterorhabditis bacteriophora* nematodes. Dotted line at 1.0 indicates normalization of fold change relative to uninfected controls. Significance levels were assessed using one-way analysis of variance (ANOVA, ****p < 0.0001).

Similar to vertebrates, intermediates of both oxygen (ROI) and nitrogen (RNO) in *D. melanogaster* constitute a prominent defense strategy against bacterial infections (33). *D. melanogaster* has a single NADPH, Dual oxidase (DUOX), which acts as a principal factor in the production of reactive oxygen species (ROS) (34, 35). Recently, it was shown that *D. melanogaster daw* mutant larvae express *DUOX* at higher levels upon infection with *H. gerrardi* nematodes (14). To elucidate whether activin and BMP branches are involved in the *D. melanogaster* DUOX mediated ROS response to *H. bacteriophora* infection, we estimated the gene expression level of *DUOX* in *daw* and *dpp* mutant larvae following infection with either axenic or symbiotic nematodes. We found no significant difference in *DUOX* expression between *daw* mutants and *w^1118^* controls when infected with either type of nematode (**Figure 3A**). Interestingly, *dpp* mutants expressed *DUOX* at higher levels compared to *w^1118^* individuals during axenic but not symbiotic *H. bacteriophora* infection. This finding indicates that the BMP branch mediates ROS response through reducing the expression of *DUOX* upon infection with *P. luminescens*-deficient *H. bacteriophora* nematodes. We have also assessed ROS levels by measuring the relative aconitase activity, yet we have not observed any significant differences between *daw* and *dpp* mutants and *w^1118^* controls upon infection with axenic or symbiotic *H. bacteriophora* (**Figure S1A**). We then investigated the association between nitric oxide (NO) response, which is controlled by the enzyme NO synthase (NOS), and TGF-ß signaling activity in the *D. melanogaster* anti-nematode response. For this, we performed qRT-PCR to determine the expression level of *NOS* and spectrophotometrically quantified nitrite protein levels in *daw* and *dpp* mutant larvae infected by either axenic or symbiotic *H. bacteriophora*. There were no statistically significant differences in *NOS* expression or nitrite protein levels between nematode infected TGF-ß mutants and the *w^1118^* controls (**Figures 3B** and **S1B**). This outcome suggests that TGF-ß signaling activity in *D. melanogaster* larvae is not involved in the NO response to parasitic nematode infection.

### Activin Signaling Activity Lowers Expression of *Diptericin* and *Cecropin* in *D. melanogaster* Larvae Following Infection With Symbiotic *H. bacteriophora*

**Figure 4:**
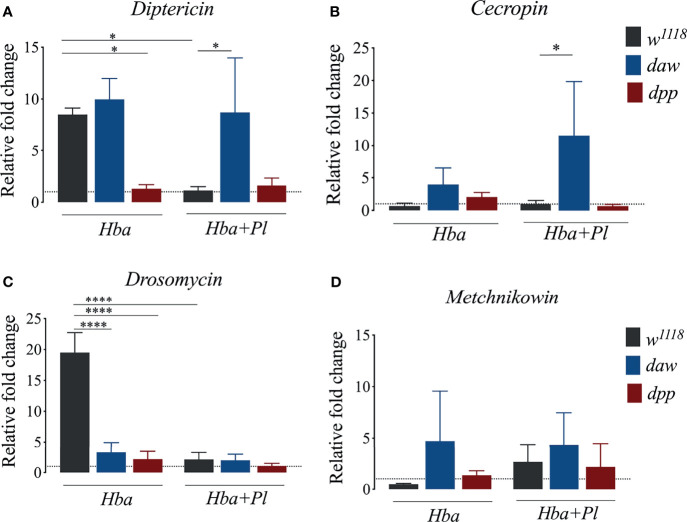
Expression of the antimicrobial peptide encoding genes *Diptericin, Cecropin*, *Drosomycin*, and *Metchnikowin* in *Drosophila melanogaster daw* and *dpp* mutant larvae and their background controls (*w^1118^*) following infection with either axenic (*Hba*) or symbiotic (*Hba*+*Pl*) *Heterorhabditis bacteriophora*. Dotted line at 1.0 indicates normalization of fold change relative to uninfected controls. Significance levels were assessed using one-way analysis of variance (ANOVA). **(A)** *Diptericin* (*p = 0.0180), **(B)** *Cecropin* (*p = 0.0318) **(C)** *Drosomycin* (****p < 0.0001), and **(D)** *Metchnikowin* expression *via* qPCR.

The hallmark of the *D. melanogaster* host defense is the inducible synthesis and secretion of antimicrobial peptides (AMPs) through NF-κB signaling pathways (36). Also, using GFP reporter transgenes, infection of *D. melanogaster* larvae with symbiotic *H. bacteriophora* results in higher percentage of fly larvae expressing the AMP encoding genes *Attacin*, *Diptericin*, *Drosomycin*, and *Metchnikowin* (19). Previous studies have demonstrated a connection between TGF-ß signaling and synthesis of AMPs in the context of parasitic nematode infection (19, 37). In addition, NF-κB/Rel signaling interferes with the expression of Activinβ (activin signaling ligand), while NF-κB/Dif signaling interferes with the expression of glass bottom boat (BMP signaling ligand) in *D. melanogaster* larvae infected by *H. gerrardi* nematodes (37). To further study the link between TGF-ß signaling activity and expression of AMPs following infection with axenic or symbiotic *H. bacteriophora* nematodes, we used qRT-PCR and gene-specific primers to determine the expression levels of *Diptericin*, *Cecropin*, *Drosomycin*, and *Metchnikowin* in *D. melanogaster daw* and *dpp* mutants. We found that following symbiotic *H. bacteriophora* infection, expression of *Diptericin* and *Cecropin* were higher in *daw* mutant larvae compared to *w^1118^* larvae (**Figures 4A, B**). *Diptericin* and *Cecropin* are mainly induced by Gram-negative bacterial infections (38). Therefore, our results indicate that these two AMPs were possibly induced by *P. luminescens* bacteria which are mutualistically associated with *H. bacteriophora* nematodes. Conversely, we found that *Drosomycin* expression significantly decreased in both *daw* and *dpp* mutants compared to *w^1118^* controls upon infection with axenic *H. bacteriophora* (**Figure 4C**). We have not found any significant differences in *Metchnikowin* expression between TGF-ß mutants and their *w^1118^* controls when infected with either axenic or symbiotic nematodes (**Figure 4D**). Taken together, these findings suggest that activin signaling activity interacts with the Imd pathway through decreasing the expression of *Diptericin* and *Cecropin* in *D. melanogaster* larvae during infection with *H. bacteriophora* nematodes harboring their mutualistic *P. luminescens* bacteria.

### Activin Signaling Activity in *D. melanogaster* Larvae Reduces the Melanization Response Upon Symbiotic *H. bacteriophora* Infection

**Figure 5:**
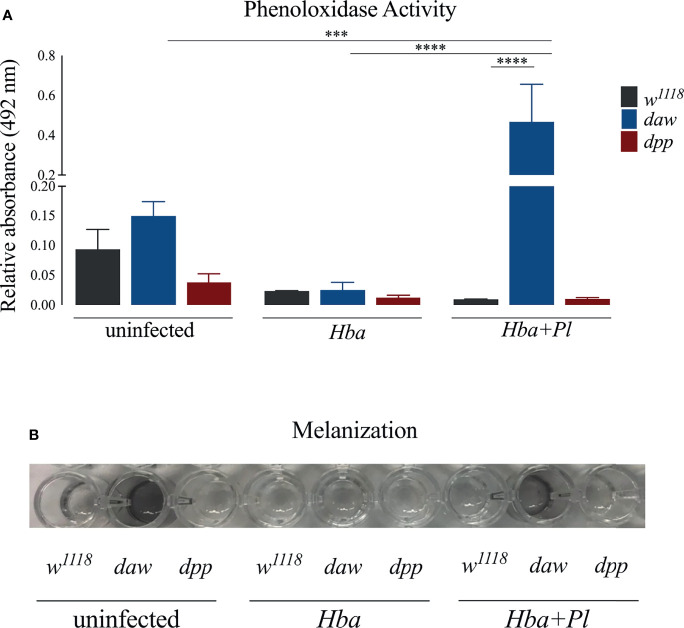
Assessment of phenoloxidase (PO) activity and melanization response in *Drosophila melanogaster* TGF-ß mutant larvae following infection with axenic (*Hba*) or symbiotic (*Hba*+*Pl*) *Heterorhabditis bacteriophora*. **(A)** PO activity in the hemolymph plasma of *daw* and *dpp* mutant larvae and their background controls (*w^1118^*) infected with either *H. bacteriophora* axenic or symbiotic nematodes. Significance levels were assessed using one-way analysis of variance (ANOVA), (***p = 0.0003; ****p < 0.0001). **(B)** Melanization of hemolymph plasma of *daw* and *dpp* mutant larvae and their background controls (*w^1118^*) infected with *H. bacteriophora* axenic or symbiotic nematodes one hour post incubation at room temperature.

Melanization mediated by the enzyme phenoloxidase (PO) is an immediate and essential response to pathogen infection in *D. melanogaster* (39). When Daw/activin signaling is impaired in the absence of infection, melanotic tumors are observed in adult flies and PO enzyme activity increases in uninfected larvae (10, 14). Interestingly, *daw* mutant larvae have lower PO activity when challenged by *H. gerrardi* nematodes, suggesting an association between the activin branch and regulation of the melanization response against parasitic nematode infection (14). To examine the interaction between TGF-ß signaling activity in *D. melanogaster* and the melanization response to axenic or symbiotic *H. bacteriophora*, we estimated the expression levels of the inactive precursors of PO *prophenoloxidase* (*PPO*) genes *PPO1*, *PPO2*, and *PPO3* in *daw* and *dpp* mutant larvae infected with either type of nematode (39). We did not observe any significant differences in *PPO1* and *PPO2* expression between the TGF-ß mutants and their *w^1118^* controls following axenic or symbiotic *H. bacteriophora* infection (**Figures S2A–C**). However, expression of *PPO3* increased significantly in *dpp* mutants compared to their background control following axenic nematode infection. The activity of PO enzyme in larval hemolymph can be measured spectrophotometrically through the detection of dopachrome which is converted by PO from L-DOPA (40). Using this method, we determined PO activity in the hemolymph plasma of *daw* and *dpp* mutants following infection with axenic or symbiotic *H. bacteriophora*. *Daw* mutants exhibited significantly increased PO activity compared to *w^1118^* controls when infected with symbiotic *H. bacteriophora*, but not with axenic nematodes (**Figure 5A**). Extraction of hemolymph followed by incubation of the plasma at room temperature leads to blackening due to the production of melanin (41). We observed that hemolymph plasma from *daw* mutant larvae, which had previously been infected with symbiotic *H. bacteriophora*, melanized at room temperature (**Figure 5B**). However, we have not observed melanization in hemolymph plasma from *dpp* mutants or background controls following symbiotic *H. bacteriophora* infection. These findings collectively suggest that activin signaling activity regulates the melanization response in *D. melanogaster* larvae through reducing the levels of PO following symbiotic *H. bacteriophora* infection.

## Discussion

**Figure 6:**
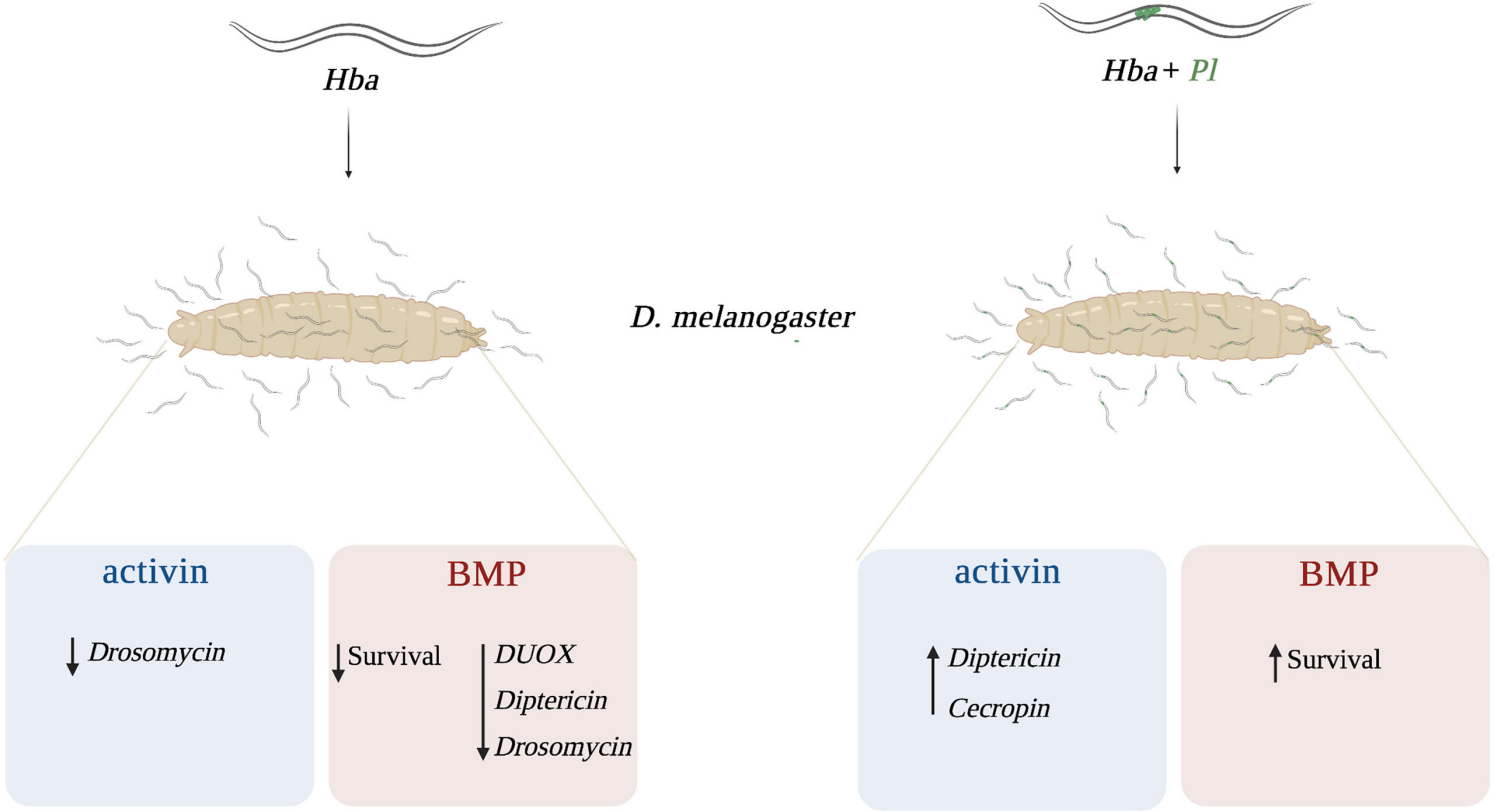
Proposed model of the interaction between activin and BMP signaling branches of the TGF-ß signaling pathway and *Drosophila melanogaster* immunity against *Heterorhabditis bacteriophora* parasitic nematodes. In response to axenic (*Hba*) *H. bacteriophora* infection, activin signaling activity reduces the expression of *Drosomycin* while BMP signaling activity reduces the survival ability and the expression of *dual oxidase* (*DUOX*), *Diptericin*, and *Drosomycin*. Following symbiotic (*Hba* + *Pl*) *H*. *bacteriophora* infection, activin signaling activity promotes the expression of *Diptericin* and *Cecropin* whereas BMP signaling activity promotes the survival of *D*. *melanogaster* larvae.

Previous studies have demonstrated that the conserved TGF-ß signaling pathway is induced in *D. melanogaster* larvae and adult flies during infection with EPNs (12, 13). Also, activin signaling activity decreases *DUOX* expression and increased PO activity in *D. melanogaster* larvae following infection with *H. gerrardi* symbiotic nematodes (14). Therefore, it is important to explore any additional roles that activin as well as BMP signaling may play in regulating cellular, humoral, and melanization immune responses in *D. melanogaster* during parasitic nematode infection. Identification of the precise molecular mechanisms underlying these processes, specifically against infection with the nematode parasites without any input from their mutualistic bacteria will contribute to a more comprehensive understanding of the host defense capability against parasitic diseases. Here, we used symbiotic together with axenic *H. bacteriophora* nematodes to examine the participation of the activin and the BMP signaling branches in the regulation of the *D melanogaster* anti-nematode immune response. We report that BMP signaling activity promotes survival against symbiotic *H. bacteriophora* infection but reduces survival against axenic nematode infection. Also, BMP signaling activity reduces *DUOX* expression but increases *Diptericin* and *Drosomycin* expression upon infection of *D. melanogaster* larvae with axenic *H. bacteriophora*. In addition, a functional activin pathway increases *Drosomycin* expression upon axenic nematode infection and decreases *Diptericin* and *Cecropin* expression upon symbiotic nematode infection. Finally, we report that activin signaling activity modulates the melanization response by reducing the PO enzyme levels upon symbiotic *H. bacteriophora* nematode parasitism (**Figure 6**).

*Heterorhabditis* nematode invasion through the cuticle causes injury to the insect host and the wounding response, which is characterized by the loss of tissue integrity, is observed throughout the infection (42). Previous work has indicated that in *D. melanogaster* larvae, *dpp* is transcriptionally induced by injury and might have a role in attenuating immune responses following wounding and infection (10). In this study, we found that *dpp* mutant larvae survive better to axenic *H. bacteriophora* infection relative to their background controls. A possible explanation for this observation could be that the injury caused by IJs during entry into *D. melanogaster* larvae, which may lead to the activation of humoral and cellular immune reactions.

Humoral immunity in *D. melanogaster* is characterized by the synthesis and secretion of AMPs into the hemolymph through the activation of two NF-κB signaling pathways, Toll and Imd (36). While the Toll signaling is predominantly activated by Gram-positive bacteria and fungi, the Imd signaling is mainly activated by Gram-negative bacteria (43–46). The Imd pathway has also been previously implicated in the immune response of *D. melanogaster* to *H. bacteriophora*. More precisely, *Diptericin*, the readout AMP-encoding gene for the Imd pathway, is upregulated at 6 h post-injection of *D. melanogaster* adult flies and larvae with excreted secreted products (ESPs) isolated from *H. bacteriophora* axenic nematodes (22, 36). In addition, feeding of *D. melanogaster* larvae with *P. luminescens* bacteria also leads to upregulation of *Diptericin* (19). Interestingly, we have found that expression of *Diptericin* is lower in *w^1118^* larvae (background control) following symbiotic *H. bacteriophora* infection compared to those infected with axenic nematodes. It is possible that at the 24 h time point, molecules produced by the nematode-bacteria pair during infection are able to suppress *Diptericin* expression. Another plausible explanation could be that *Diptericin* expression starts to decrease in larvae at 6 h post symbiotic *H. bacteriophora* infection, similar to *Diptericin* expression decrease in adult flies infected with *Escherichia coli* or *Micrococcus luteus* (47). We also demonstrate that *Diptericin* and *Cecropin* are upregulated following symbiotic *H. bacteriophora* infection in activin deficient larvae compared to the background control line. This implies that activin signaling activity interacts with the Imd pathway in *D. melanogaster* larvae during infection with the *H. bacteriophora-P. luminescens* complex to modify the AMP response. However, additional work is required to fully elucidate if Imd and activin signaling in *D. melanogaster* work synergistically against parasitic nematode infection. It would be interesting to distinguish the factors produced by the nematodes and their mutualistic bacteria to determine those that lead to *Diptericin* upregulation in *daw* mutants. The two virulence factors secreted by *H. bacteriophora* nematodes, Ecdysteroid glycosyltransferase Hba_07292 and Serine carboxypeptidase (Hba_11636) are able to suppress *Diptericin* expression in *D. melanogaster* larvae (40, 48). Investigating whether injection of these factors into *daw* mutant larvae is associated with any changes in *Diptericin* expression would provide insight about the impact of ESPs on activin-Imd interaction.

Previous work has shown that in the absence of infection, BMP signaling is regulated by Toll signaling activity *via* binding of Dorsal to *Dpp* and suppressing its expression in the ventral domain of the embryo (49). The interplay between these two signaling pathways is essential for *D. melanogaster* development (50). Here, we suggest that interaction between TGF-ß signaling branches and Toll signaling could be further extended to the immune response against parasitic nematode infection. This is particularly due to our observation of the reduced expression of *Drosomycin* in both *daw* and *dpp* mutants upon axenic *H. bacteriophora* infection. Our findings provide a basis for future investigations aimed at elucidating interactions between activin and BMP signaling activity with the Toll pathway during the *D. melanogaster* response to *H. bacteriophora*. Toll signaling receptors, GNBP1, PGRP-SA and PGRP-SD are upregulated following infection with symbiotic *H. bacteriophora* nematodes in *D. melanogaster* adult flies (36, 51). To identify potential interactions between TGF-ß and Toll signaling at the level of recognition, changes in the expression of *daw* and *dpp* following infection with *H. bacteriophora* nematodes or injection with ESPs can be studied in flies deficient of GNBP1, PGRP-SA or PGRP-SD. Moreover, to identify the interactions at downstream expression, flies deficient of the Toll pathway transcription factor Dif could be used to assess the expression of *daw* and *dpp* or other intracellular TGF-ß signaling components in the context of response to parasitic nematode infection (36). Lessons we learn through studying these interactions could be applicable to higher organisms, including humans, considering the similarities between innate immunity in *D. melanogaster* and mammals and the phylogenetic relationship between *H. bacteriophora* nematodes and vertebrate parasitic nematodes including *Necator*, *Dictyocaulus*, and *Oslerus* (52–54).

In our study we found that *D. melanogaster* activin deficient larvae have increased levels of PO as well as melanin formation in their hemolymph plasma compared to their background controls following infection with symbiotic *H. bacteriophora*. This finding suggests that activin signaling activity reduces the melanization response in *D. melanogaster* larvae upon symbiotic nematode infection. In contrast, we have not found any significant differences in *daw* mutants following axenic *H. bacteriophora* infection suggesting that activin signaling activity reduces the melanization response only when nematodes infect *D. melanogaster* larvae together with their mutualistic bacteria. Interestingly, a recent study has shown that following infection of *daw* mutant larvae with symbiotic *H. gerrardi* nematodes, PO activity remains at low levels (14). It is therefore possible that the property of functional activin signaling to decrease the melanization response in *D. melanogaster* larvae is restricted only to the response against the *H. bacteriophora*-*P. luminescens* complex. It would be interesting to further explore how other parasitic nematodes influence the interaction between activin signaling activity and the melanization response by testing the response of *D. melanogaster* to other EPNs such as *S. carpocapsae*. Infection of *D. melanogaster* larvae with *S. carpocapsae* nematodes leads to increased PO activity in the hemolymph (23). In contrast, inoculation of the *S. carpocapsae* mutualistic bacteria, *Xenorhabdus nematophila*, *via* pricking into *D. suzukii* larvae results in lower PO activity in the hemolymph (55). Based on this information, it would be interesting to test whether activin signaling activity has a role in regulating the *D. melanogaster* PO activity and melanization response against the *S. carpocapsae-X. nematophila* complex. In addition, considering the role of melanization in clot formation, another field of inquiry would be to study whether activin signaling interacts with the clotting response in *D. melanogaster* against parasitic nematode infection (56). Clotting factors such as Fondue, Eig71Ee, and transglutaminase protect *D. melanogaster* larvae against symbiotic *H. bacteriophora* infection (57, 58). Future studies could focus on understanding if this protection extends to the mutants of activin signaling suggesting an interaction between clotting and TGF-ß signaling activity during the *D. melanogaster* response to EPN infection. This is a particularly interesting avenue of research because not only it can provide important insight about the regulation of clotting response in mammals against infection with nematode parasites but also it can give clues about the evolutionary basis of anti-parasitic immune response.

Although we cannot exclude the possibility that the current observations may be due to the different genetic background of the mutants as compared to the control flies and rescue experiments may be needed to confirm the observed phenotypes, our findings demonstrate novel functions for activin and BMP signaling activity in regulating the *D. melanogaster* immune response to parasitic nematode infection. In addition to paving the way to a better understanding of host-parasite interactions and the evolution of the immune role of TGF-ß signaling, results obtained from this study could have broader impacts. Parasitic nematodes pose a major threat to human health, and thus identification of the key immune signaling components that oppose infection against parasitic nematodes will provide important clues for the development of novel treatment strategies against parasitic diseases.

## Data Availability Statement

The original contributions presented in the study are included in the article/**Supplementary Material**. Further inquiries can be directed to the corresponding author.

## Author Contributions

YO designed and conducted the experiments, analyzed the data, constructed the figures, interpreted the results, and wrote drafts of the manuscript. DR conducted parts of the experiments. IE designed the experiments, interpreted the results, and revised the manuscript. All authors contributed to the article and approved the submitted version.

## Funding

This research was funded by the National Institute of Allergy and Infectious Diseases (grants 1R01AI110675 and 1R56AI110675) and the National Science Foundation (grant 2019869).

## Conflict of Interest

The authors declare that the research was conducted in the absence of any commercial or financial relationships that could be construed as a potential conflict of interest.

## Publisher’s Note

All claims expressed in this article are solely those of the authors and do not necessarily represent those of their affiliated organizations, or those of the publisher, the editors and the reviewers. Any product that may be evaluated in this article, or claim that may be made by its manufacturer, is not guaranteed or endorsed by the publisher.
