# Supplementary material for: Activin and BMP Signaling Activity Affects Different Aspects of Host Anti-Nematode Immunity in Drosophila melanogaster
Source: Front Immunol. 2021 Dec 22;12:795331. doi: 10.3389/fimmu.2021.795331 (PMC8727596; doi:10.3389/fimmu.2021.795331)
Supplement: Supplementary file 1 [file DataSheet_1.docx]

Supplementary Material

**
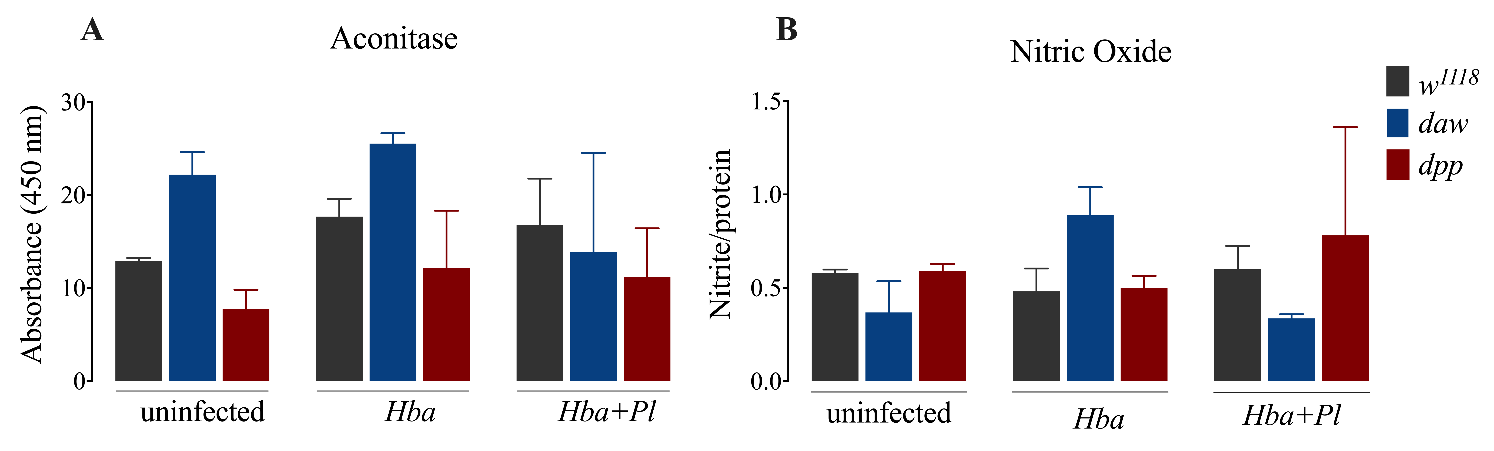
**

**Supplementary Figure 1 (A)** Aconitase and **(B)** nitric oxide (NO) levels in *Drosophila melanogaster* *daw* and *dpp* mutants following infection with either axenic (*Hba*) or symbiotic (*Hba*+*Pl*) *Heterorhabditis bacteriophora* parasitic nematodes. Significance levels were assessed using one-way analysis of variance (ANOVA). P values of >0.05 were considered non-significant.


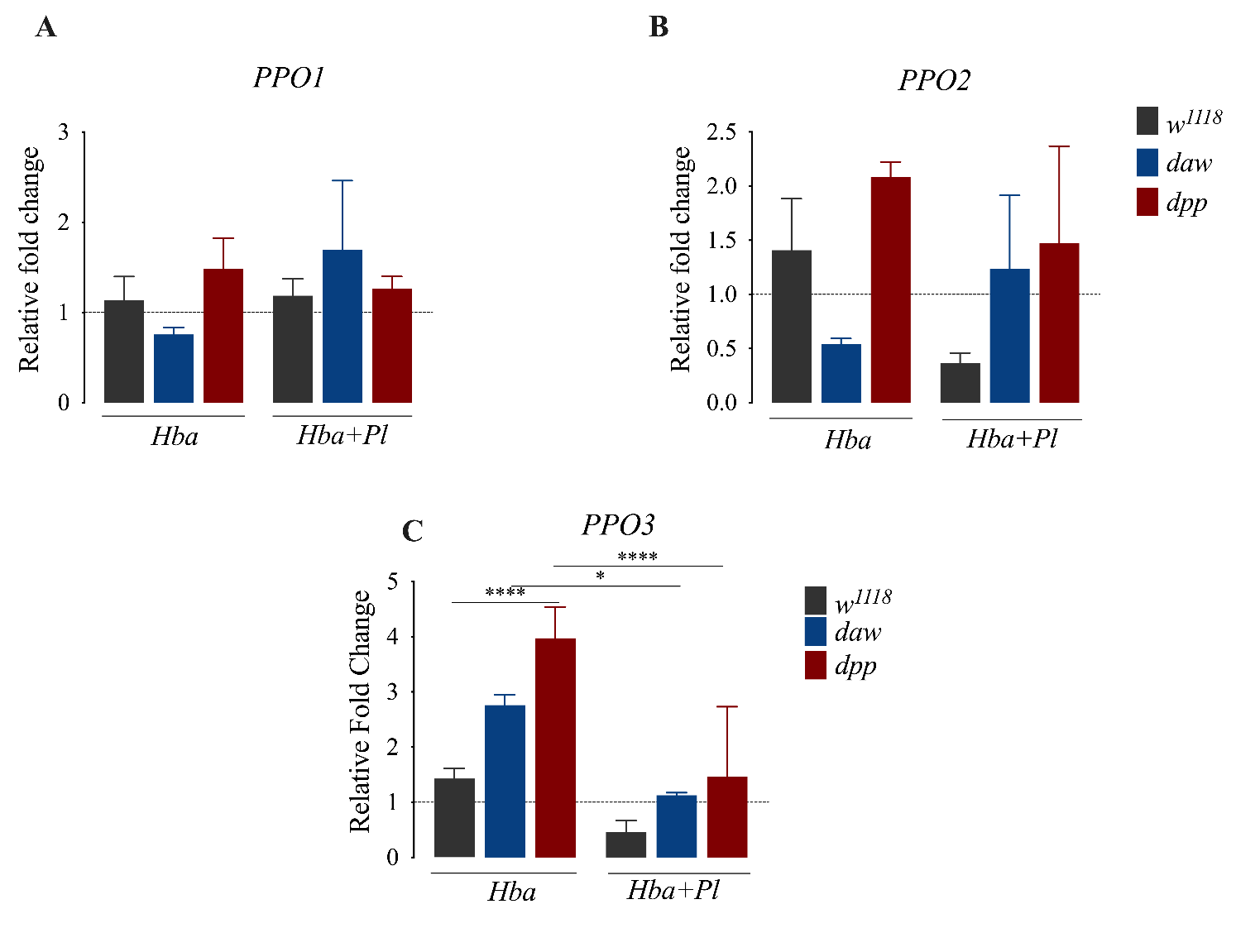


**Supplementary Figure 2. (A)** Quantitative PCR analysis of *prophenoloxidase 1* (*PPO1*) and **(B)** *prophenoloxidase 2* (*PPO2*) and **(C)** *prophenoloxidase 3* (*PPO3*) expression in *Drosophila melanogaster* *daw* and *dpp* mutant larvae and their background controls (*w^1118^*) responding to infection with either axenic (*Hba*) or symbiotic (*Hba*+*Pl*) *Heterorhabditis* *bacteriophora* nematodes. *﻿*Dotted line at 1.0 indicates normalization of fold change relative to uninfected controls*.* **(C)******p<0.0001; *p= 0.0237. Significance levels were assessed using one-way analysis of variance (ANOVA). ﻿P values of >0.05 were considered nonsignificant.
